# EMPATHIC-N in a Greek-Cypriot sample: confirming its factorial structure

**DOI:** 10.1186/s12913-018-3793-3

**Published:** 2018-12-14

**Authors:** Elena Papamichael, Myria Ioannou, Michael A. Talias

**Affiliations:** 1 Neonatal Intensive Care Unit, Arch. Makarios III Hospital, 6 Koritsas street, 1474, Strovolos, Nicosia, Cyprus; 2 Psychology Department, University of Cyprus, Nicosia, Cyprus; 3 Health Management Unit, Open University of Cyprus, Nicosia, Cyprus

**Keywords:** Parental satisfaction, Neonatal intensive care, Validation, Confirmatory factor analysis

## Abstract

**Background:**

Family-centered care has been receiving increased attention during the last decades and health professionals recognize family satisfaction with care as an important health indicator. The Empowerment of Parents in The Intensive Care-Neonatology (EMPATHIC-N) is a newly developed, yet empirically reliable and valid measure for the assessment of parental satisfaction with the care provided by Neonatal Intensive Care Units (NICU). The present study aims to present the Greek version of the EMPATHIC-N and to confirm its factorial structure.

**Methods:**

The EMPATHIC-N was translated in Greek using a forward-backward translation and was piloted before use. A sample of 256 families receiving intensive care at the NICU of Archbishop Makarios III Public Hospital in Cyprus which is the only NICU in Cyprus, participated in the validation study of the EMPATHIC-N. Confirmatory factor analyses were performed using SPSS and AMOS 24.0.

**Results:**

The Greek version of the EMPATHIC-N had good psychometric characteristics (Cronbach’s alpha = .87). The CFAs for the separate subscales of professionalism, organization, information, parental involvement and intervention for the EMPATHIC-N showed that all five subscales represented five distinct components of parental satisfaction with care. The CFA of the general instrument supported that a second-order model with a higher-order factor reflecting the organizational structure (professionalism, intervention and organization loaded on this factor) fitted the data best [χ2 (259) = 405.332, *p* < .001, ΤLI = .887, CFI = .903, RMSEA = .065 (90% CI .058, .073), SRMR = .0597].

**Conclusions:**

EMPATHIC-N is a valid and reliable measure for the assessment of parental satisfaction with neonatal care in a Greek-Cypriot context. The organizational dimension of the NICUs is an important component with specific research and clinical implications for the enhancement of parental satisfaction with care.

**Electronic supplementary material:**

The online version of this article (10.1186/s12913-018-3793-3) contains supplementary material, which is available to authorized users.

## Background

Family-centered therapy is defined as a specialized procedure in the assessment, treatment and care of babies, infants and adults and is established through the collaboration between providers of care and family. This care approach is used in Australia, Canada, United Kingdom, Island, Sweden, USA [1–3]. Family-centered therapy presupposes the exchange of information related to children’s care between family and care providers and the development of the sense of having some control on medical choices by the family. In this framework, family is considered as the primary source of strength and support during children’s treatment; therefore family-centered therapy promotes services characterized by flexibility and empowerment of the family [4]. Decreased length of stay, development of secure parent-infant attachment, improved medical state and general well-being of preterm infants, better allocation of care resources, and high patient and family satisfaction are only some of the benefits reported when implementing family-centered therapy [5, 6].

In this framework, the formation of family satisfaction questionnaires with medical care is very important, as parental satisfaction can be used as a quality of health indicator [7]. Parents ask for the best available care for their newborns and during their stay at the Neonatal Intensive Care Units (NICU) may need emotional empowerment and increased provided information in order to handle their baby’s problem. As a result, the report of parental experiences and their satisfaction concerning the care provided is a useful tool, a quality measure and a way to improve [8–10].

The Empowerment of Parents in The Intensive Care-Neonatology (EMPATHIC-N questionnaire) by Latour and associates [11] is considered to be a valid indicator to measure parental satisfaction with health services. At first, Latour and associates [12, 13] created an instrument for measuring parental satisfaction for children admitted in Pediatric intensive care units (PICUs), namely EMPATHIC questionnaire [13]. This questionnaire consisted of 65 items which loaded on five domains and its structure was confirmed using confirmatory factor analysis as well. This was performed in two separate studies which recruited population from 8 PICUs in the Netherlands. A cohort of 220 parents took part in the first study and 59 parents participated in the second one. The exploratory factor analysis showed that the loadings for all items were > 0.40. Additionally the questionnaire had acceptable internal consistency, as Cronbach’s a ranged from 0.73 to 0.93 for each subscale.

The increasing demand to provide care based on consumers’ reviews, results in the need to accept that patient satisfaction stands out as a significant quality performance measure. Therefore, the development of parental satisfaction instruments for the care provided by health professionals to newborns is mandatory [14, 15]. The EMPATHIC-N (Empowerment of Parents in The Intensive Care-Neonatology) questionnaire [11] was used in two cohorts consisting by 441 parents who had a newborn admitted in a neonatal intensive care unit. The participation reached 65 and 58% in each cohort, respectively. The exploratory and confirmatory factor analyses showed that the 57 statements of the questionnaire were loading on five subscales: Information, Care and Treatment (Ιntervention), Parental Participation, Organization, and Professional Attitude. The standardized factor loadings of the items on the subscales ranged between 0.58–0.91, and the reliability of the subscales ranged between Cronbach’s a = 0.82–0.95.

EMPATHIC-N was suggested by Latour and associates [11] to be a valid measure of the quality of delivered care as understood and reported by parents. Dall’ Oglio and associates [16] have translated and used the EMPATHIC-N in an Italian study aiming to measure parental satisfaction. The EMPATHIC-N has also been translated and used in Brazil [17]. The psychometric properties of the questionnaire were confirmed in both countries.

The EMPATHIC-N is nowadays one of the most popular instruments measuring parental satisfaction [18]. The development of the EMPATHIC-N is based on the theoretical notions that through the sharing of information, professionals and parents are getting to know each other and begin their collaboration for the care of the newborn child. The effective and understanding communication between parents and professionals can be supportive and helpful for the child, and is able to decrease parental stress due to the trust developed between parents and professionals. Providing information and educating parents for the procedures followed by the NICU is considered a major challenge for health professionals [11]. Intervention and care are also considered two of the most important dimensions for the satisfaction of patients [19]. Immediate intervention in the case that the health situation of a newborn child gets aggravated, the knowledge of the full health history of the newborn, the provision of the correct treatment, the prevention and treatment of pain, the correct time of providing a treatment, the close collaboration of health professionals and the support of the family as a system are major components of the parental satisfaction regarding intervention and care [11]. At the same time, the flexibility on the visiting hours in NICU, the participation in newborns’ care and the parental involvement in the important decisions for the progress of a newborn’s health are significant components of parental satisfaction. Even though research accepts that medical staff often has difficulties in the communication with the parents, there is a general approval and necessity of family-centered therapy which respects parental involvement. According to Azoulay and colleagues [20], medical staff has to invest and give fundamental support to the parents, having in mind that this support empowers and increases their expectations. Family-centered care in NICUs as a procedure isn’t new, but there’s a lot to be done to improve it.

Health care systems today are characterized as complex, in flux, technically proficient, competitive and market-driven. Strong emphasis is being placed on customer service, with efforts to understand, measure and report the needs of consumers being served. Patient satisfaction is rapidly becoming a primary indicator for evaluation and comparison of quality in health care plans. This model suggests that patient satisfaction represents a strong quality measure [21]. Patient satisfaction can be influenced by the organizational standards of the hospital units, the attitude of medical and nursing staff and the quality of information given. These structural components in health organizations increase parental commitment in their newborns’ care and aim on the construction of a family-centered environment with high standards of care [7]. The need to translate questionnaires to assess quality of health care provided in NICUs is underlined. The absence of suitable and valid measures in Greek language results in unknown levels of patient satisfaction with NICU [22], and hence limited efforts to change current satisfaction levels by improving health care provided to newborns.

### The present study

Cyprus is a small independent island in the Mediterranean. The NICU is the only 3rd level unit in the country taking care of all neonates admitted from private and government sector. Therefore, NICU’s epidemiological findings present a realistic image of perinatal health in Cyprus and this is noted also by the Cyprus Ministry of Health [23]. So far, there have not been any systematic evaluations of the level of parental satisfaction in the NICU, though they could have been used as a quality measure of the provided services. Nowadays this knowledge is very important for the health organizations to further improve and also in order to attract financial institutions to invest money in health systems, in research and innovation. The present study aims to a) examine the psychometric properties of the EMPATHIC-N in Greek using a Greek-Cypriot population, b) test the structural conceptualization of parental satisfaction by comparing models based on the confirmatory factor analyses of the EMPATHIC-N. To our knowledge, this is the first study that attempts to translate the EMPATHIC-N in Greek and to confirm its factorial structure. Previous examination of the structure of EMPATHIC-N using confirmatory methods concerned the analysis of each subscale separately [13], but a concurrent confirmatory factor analysis of the whole instrument has not been tested before.

A previous adaptation of the instrument in Brazilian Portuguese did not include confirmation of the questionnaire’s structure [17]. A recent adaptation of the instrument in Italian used confirmatory factor analysis on the whole questionnaire but did not include a step-by-step confirmatory analysis of each subscale [24]. Also, the correlations between the first-order latent factors of the model were not provided, and the feasibility of a second-order analysis was not supported. Therefore, the present study aims to test the factorial structure of the Greek version of the EMPATHIC-N, using confirmatory factor analyses for each subscale and then for the whole instrument. The translation of the EMPATHIC-N in Greek was considered important, due to the absence of any available questionnaires assessing the quality of health provided by NICUs in Cyprus. At the same time, confirmation of the factorial structure of the original EMPATHIC-N was of substantial significance, due to the need to generalize the domains affecting parental satisfaction with the NICUs worldwide and the enhanced communication of clinicians and researchers working with this population. Confirmatory factor analyses are considered important for hypothesis testing and for the replication of the factorial structure of an empirically valid questionnaire. Due to the options to use CFA as a strictly confirmatory procedure, as an alternative model exploration, or as a model generation technique, this approach is preferred over the EFAs [25]. Specifically, we aimed to compare different models for the factorial structure of the EMPATHIC-N. Firstly, we aimed to test the existence of five latent factors and the correlations between them. At the same time, we aimed to examine second-order models if founding high correlations between the first-order latent factors of information, organization, intervention, professionalism and parental involvement. This would allow not only a confirmation of the factorial structure of the original instrument, but would also provide more theoretical and practical implications regarding the interactions between the dimensions determining parental satisfaction with NICU care. Replication of the factorial structure of the Greek-version of the EMPATHIC-N using exploratory and confirmatory methods would stand as evidence for its psychometric adequacy.

## Methods

### Participants

Two hundred fifty-six families from a larger sample of 640 families that were hospitalized in the Neonatal Intensive Care Unit (NICU) in Cyprus between March 2013 and March 2014 participated in the present study. The sample of 256 families reflects the sample that gave consent for participation in the study. The present study is part of a larger project investigating the epidemiology of prematurity and characteristics that could predict it, in which the total number of families hospitalized was used for data records. The present study constitutes part of the research project done by the first author in fulfillment of PhD requirements at the Open University of Cyprus. One of the parents of the newborns had to complete the Greek version of the EMPATHIC-N questionnaire at the end of their stay in the NICU. We did record some demographic characteristics of the parents who completed the questionnaire (Table 1). Based on these, the sample seemed representative of the socio-economic and demographical background of the general population. As for the newborns’ gender, 52.4% were males and 47.6% were females. Among the sample of 256 families (one parent in each case) who participated in the study, 3 parents provided less than 25% of responses in the questionnaire and were excluded from analyses. Among the rest, a missing value analysis showed that the missing value percentage was less than 2%. As the values were missing at random, they were imputed using a regression-based estimation. Sensitivity analyses were performed using the dataset with missing values and the option ‘Estimate means and intercepts’ in AMOS, in order to examine the stability of the findings stemming from the dataset with the imputed values.

**Table 1:** Socio-demographic information recorded for parents

Demographic variables	Ν (%)
Maternal educational level	
Primary school	2 (0.8)
Middle school	12 (4.8)
High school	67 (27.0)
College degree	50 (20.2)
University degree	117 (47.2)
Paternal educational level	
Primary school	10 (4.0)
Middle school	19 (7.7)
High school	97 (39.3)
College degree	35 (14.2)
University degree	86 (34.8)
Maternal origin	
Greek-Cypriot	219 (85.5)
Turkish-Cypriot	2 (0.8)
Maronite	1 (0.4)
European Union citizen	26 (10.2)
Other	5 (2.0)
Armenian	1 (0.4)
Paternal origin	
Greek-Cypriot	221 (86.3)
Turkish-Cypriot	2 (0.8)
Maronite	1 (0.4)
European Union citizen	21 (8.2)
Other	5 (2.0)
Armenian	1 (0.4)
Maternal employment status	
Self-employed	20 (8.3)
Governmental/public employee	55 (22.7)
Private employee	95 (39.3)
Half-governmental employee	22 (9.1)
Non-employed	43 (17.8)
Paternal employment status	
Self-employed	39 (16.1)
Governmental/public employee	48 (19.8)
Private employee	118 (48.8)
Half-governmental employee	9 (3.7)
Non-employed	27 (11.2)
Maternal income (euro per year)	
< 10,000	73 (34.6)
10,000–20,000	75 (35.5)
20,000–30,000	53 (25.1)
30,000–40,000	7 (3.3)
> 40,000	3 (1.4)
Paternal income (euro per year)	
< 10,000	55 (24.3)
10,000–20,000	97 (42.9)
20,000–30,000	53 (23.5)
30,000–40,000	18 (8.0)
> 40,000	3 (1.3)
Family status	
Divorced	6 (2.4)
Married	234 (92.5)
Total number of kids	
1	96 (37.5)
2	102 (39.8)
3	43 (16.8)
4	11 (4.3)
5	1 (0.4)
6	1 (0.4)

### Procedure

The present study was conducted at the Neonatal Intensive Care Unit of Archbishop Makarios III Public Hospital in Cyprus. The data collection of the study lasted one year (March 2013 – March 2014). Before conducting the research, we received approval by the Ministry of Health (no of license: 5.34.01.7.2E, protocol: 0080/2013), by the head of the Pediatric/NICU Clinic, by the hospital’s Chief Medical officer and lastly by the Cyprus Bioethics Committee (ΕΕΒΚ ΕΠ2013.01.09). We recorded all the cases which were treated at NICU during those 12 months.

The families that took part in the study participated voluntarily and no inclusion criteria were set for their participation. We firstly received license to use the EMPATHIC-N by the developer of the instrument Dr. J. Latour, and then the questionnaire was translated following a forward-backward translation method. The syntactical structure of the questionnaire and the vocabulary used were evaluated and accepted by two independent researchers (third author M.T., and one colleague from the NICU). The questionnaires were placed in sealed envelopes and were given to parents for completion before discharge, who returned the completed questionnaires in the same way to the first author (EP).

### Instruments

The EMPATHIC-N consists of 57 self-report questions loading on five factors: the *information* which parents receive for their hospitalized newborn child, the *intervention and care* that their newborns receive, the *parental involvement* reinforced by the NICU, the *organization* of the NICU and the *professional behavior* of the NICU staff. The 57 items are answered on a 5-point scale ranging from 1 = never to 5 = always. The EMPATHIC-N is presented in Additional file 1 and a short description of each of the five subscales follows below:

The dimension of Information consists of 12 questions and refers to the details provided to parents regarding the decision made and care concerning their child. Intervention and care dimension includes 17 items and reflects the parental satisfaction with the care provided in the NICU. In this questionnaire, parental involvement is defined by 8 items which reflect the degree at which parents are allowed and reinforced to get involved in the care of their newborns and in decisions that concern them. Organization is considered a basic domain in health services. This subscale consists of 8 questions that concern the need for easy communication with the people working in NICU, the clean environment, the feeling of hospitality and safety, the large spaces, the low noise and other organizational characteristics. The last component reflects professionalism behavior and this is examined by 12 questions emphasizing on the respect in different cultures, the discipline in hygiene rules, the individualized given care and the respect of every family’s privacy.

### Statistical analysis

Data were entered in SPSS Version 20.0 which was used for statistical analyses at a descriptive level. Reliability analyses were conducted using SPSS, whereas confirmatory factor analyses were tested using AMOS 19.0.

The five latent factors of the parental satisfaction dimensions were reflected by the respective number of observed variables /items loading on each subscale (described in the instruments section above). We tested the factorial structure for each one of the five subscales from the parental satisfaction scale separately, and then all items were included in the same model. The missing values percentage was lower than 2% and the data missing were missing at random. Therefore, missing values were replaced by regression estimation, based on the responses of parents in the rest of the questions from each subscale. The estimation method was maximum likelihood (MLE), as determined by the univariate and multivariate normality of the data. For the assessment of the model fit for each model, we evaluated the following indices: chi square test (CMIN, p-test non-significant), Comparative fit index (excellent fit CFI > .95, acceptable fit CFI > .90), Tucker-Lewis index (excellent fit TLI > .95, acceptable fit TLI > .90), Root mean square error of approximation RMSEA (excellent fit < .05, acceptable fit < .08), and Standardized Root Mean Square Residual (excellent fit < .05, acceptable fit < .08). Most model fit indices should be acceptable in order to evaluate a CFA model as acceptable, based on the suggested criteria provided by Hu and Bentler [26].

## Results

All subscales had high internal consistency as evident by the Cronbach’s alpha (Table 2) and the general scale had high internal reliability with a = .87.

**Table 2:** Fit indices of the Confirmatory Factor Analyses for the five factors of parental satisfaction

Confirmatory Factor Analyses Models	Chi square (df)	*P* value	CFI	TLI	GFI	RMSEA (90% CI)	SRMR	Cronbach’s alpha
Organization (8 items)	47.283 (19)	< .001	.946	.921	.960	.075 (.049, .104)	.0431	.744
Parental involvement (8 items)	32.870 (16)	.008	.981	.966	.969	.064 (.032, .096)	.0357	.841
Intervention (17 items)	183.578 (71)	< .001	.930	.910	.907	.079 (.065, .093)	.0541	.863
Information (12 items)	113.795 (50)	< .001	.941	.923	.926	.071 (.054, .088)	.0530	.820
Professionalism (12 items)	134.547 (51)	< .001	.933	.913	.921	.080 (.064, .097)	.0490	.871
Model A (5 inter-correlated factors)	505.632 (255)	< .001	.898	.913	.917	.062 (.054, .070)	.0535	
Model B (with second-order factor)	405.332 (259)	< .001	.887	.903	.912	.065 (.058, .073)	.0597	

**Fig. 1:**
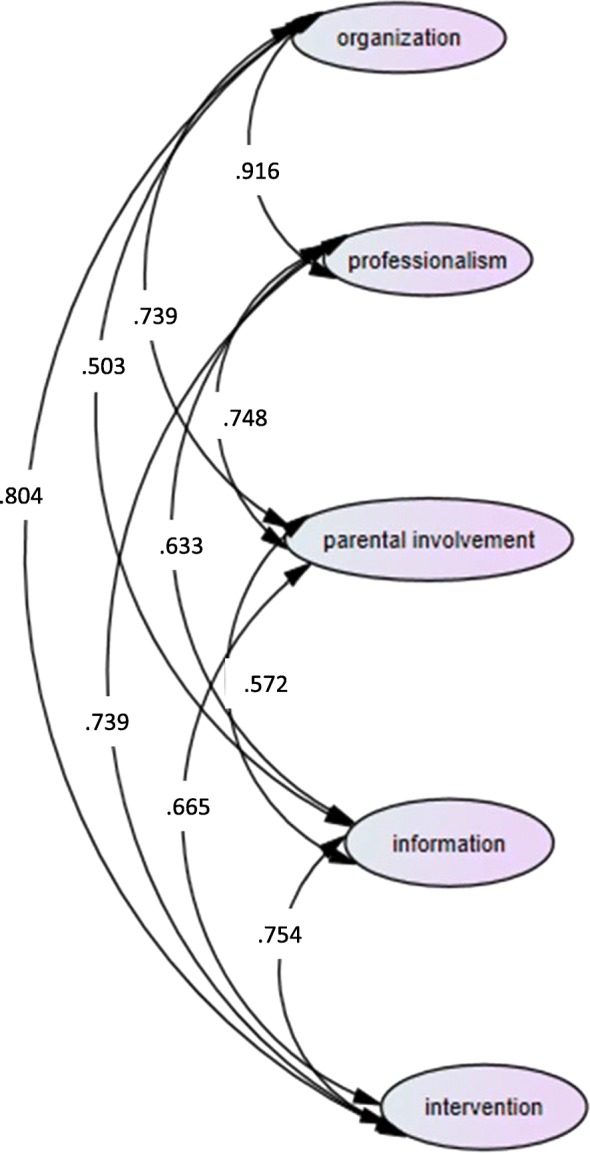
Confirmatory Factor Analysis for the five factors of parental satisfaction. The estimates presented are the standardized estimates

In order to test the confirmatory factor analysis of the EMPATHIC-N, we examined the confirmatory factor analyses of each subscale separately. All the subscales had acceptable model fit, as shown on Table 2. Then we inserted all the items as observed indicators, loading on the five latent factors of information, intervention, organization, parental involvement and professionalism. The model had acceptable model fit indices, with χ2 (255) = 505.632, *p* < .001, ΤLI = .898, CFI = .913, RMSEA = .062 (90% CI .054, .070), SRMR = .0535. The model with the standardized estimates of the interrelations between the latent factors is presented in Fig. 1. The examination of the correlation magnitudes showed than not all of the correlations between the five factors were strong, in order to justify the existence of a general factor of satisfaction with care (i.e., second-order factor). However, the correlations between the intervention, the organization and the professionalism were considerably high (two of them higher than >.80), therefore justifying the examination of a second-order CFA.

**Fig. 2:**
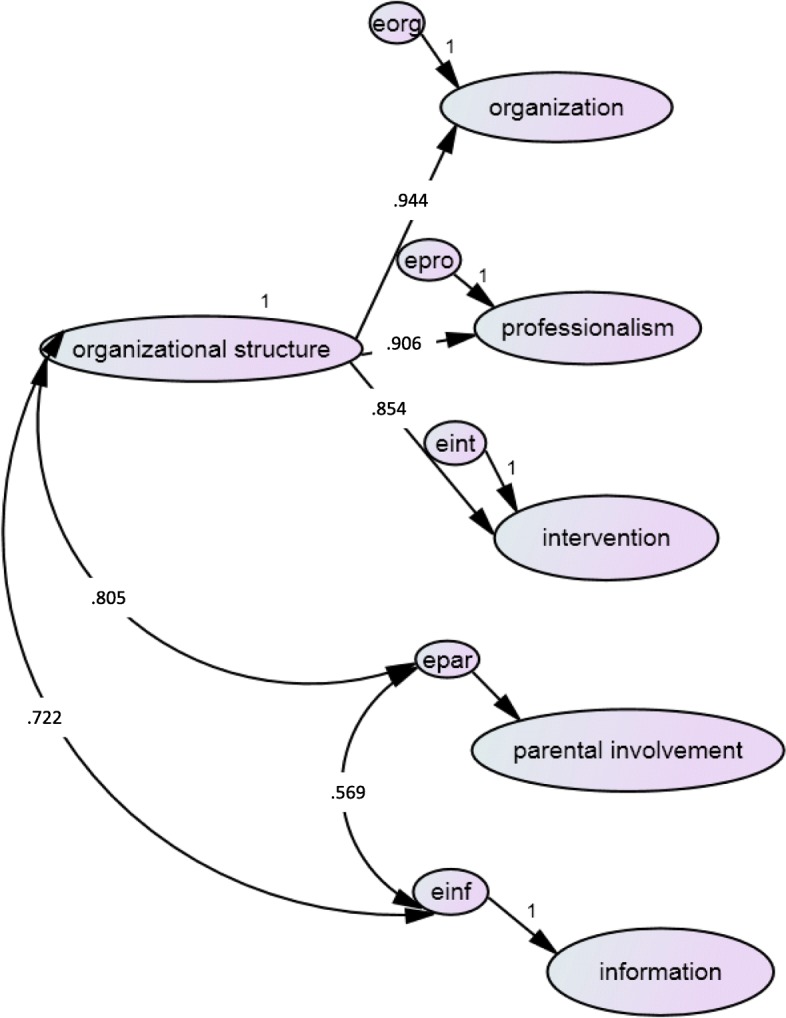
Second-order Confirmatory Factor Analysis Model (chosen model based on the fit indices)

The second-order CFA (Fig. 2) showed that the first-order factors of intervention, professionalism and organization had significant standardized estimates of high magnitude on the second-order factor (> .854), confirming the appropriateness to load on a second-order factor together. The second-order factor seemed to reflect the organizational structure, as it included the roles of health professionals, whereas the other two factors which did not show to have high correlations with the rest seemed to reflect the interaction between parents and health professionals which was not clearly related to organizational grounds (i.e., parental involvement, information). The second-order CFA was found to have acceptable fit indices, with χ2 (259) = 405.332, *p* < .001, ΤLI = .887, CFI = .903, RMSEA = .065 (90% CI .058, .073), SRMR = .0597. The correlations between the factors of parental involvement and information with the second-order factor of organizational structure were high (*r* = .805 and *r* = .722, respectively). The factors of information and parental involvement had a positive and high correlation between them, but not as high (>.80) to suggest the existence of a second-order factor linking the two. The second-order factor model had statistically significantly better model fit compared to the five correlated factors model, with Δχ^2^ (4) = 100.300, *p* < .001.

## Discussion

The present study examined the factorial structure of the Greek version of the EMPATHIC-N. The findings supported significant loadings of the corresponding items on the five subscales consisting the instrument. Separate confirmatory factory analyses for each subscale supported acceptable models for all subscales. The inclusion of all subscales into one single CFA supported a higher-order model with an extra relation between the factors of intervention, organization and professional behavior. Even though parental involvement and information provided to parents had generally high correlation with the organizational structure dimension, they seemed to capture slightly different constructs of parental satisfaction.

Our findings are in line with previous investigations of the adaptation of EMPATHIC-N in other languages. It seems that this instrument can be a generalizable and easily understood instrument of parental satisfaction that can be adapted in different cultural and linguistic backgrounds. However, our findings regarding CFA are not in line with the single existing investigation of EMPATHIC-N using CFA which was done by Dall’Oglio and colleagues [24]. The authors of the Italian adaptation of EMPATHIC-N have supported a second-order factor of the instrument, on which all five subscales loaded significantly and captured the general concept of parental satisfaction with care. The results of the present study pointed to a special relation between the organizational dimensions that constitute parental satisfaction, suggesting that these factors represent a distinct identifiable concept when parents rate their satisfaction with neonatal care. This has specific implications for research and clinical practice.

Firstly, research on parental satisfaction with care needs to pay special attention on the examination of the organizational structure characteristics and the related socio-demographic and care-related factors that predict parental satisfaction with the organization of the neonatal care unit. Even though the present study did not attempt to examine the degree of parental satisfaction with the NICU, nor the quality of health care provided in the NICU per se, the conceptualization of the organizational structure as a common construct reflected through organization, professional behavior and parental involvement outlines the importance of this construct. At the clinical level, health professionals working with sensitive populations in the NICUs should have structured ways to involve parents in the care of their newborns by informing them and letting them get involved with decisions concerning the interventions employed. Importantly, the professionalisms’ behavior at the person-level and the unit-level should enhance parental trust with neonatal care provided, though the provision of organizational structure and well-planned and evidence-based care. Involving parents in the NICUs should be seen as the state of the art and a way to make health professionals feel more comfortable when interacting with parents; and not as a space for criticism and judgmental approaches from parents to health professionals.

In our study, parental involvement and information were highly correlated with the unit’s organizational structure, but did not reflect the exact same construct. Theoretical implications of the study include the need to conceptualize organizational structure as a basic component in models concerned with parental satisfaction with care. Organizational structure in health systems is reflected through the professional attitude of the medical staff towards parents and is evident through all the procedures that include parents in their newborns’ care. The practical implications of this study indicate the importance of promoting and enhancing the organizational structure of the unit by educating the medical and nursing staff to maintain professional manners and provide to parents the necessary information about the intervention and any medical decisions that need to be taken in an understandable way. The medical and nursing staff need to find ways to communicate this organizational structure with the parents, while keeping them empowered enough to be involved in their newborns’ care. Recent approaches in the NICU emphasize the need to more actively include parents in care while at the same time supporting them emotionally: for example, Hall and colleagues [27] have suggested the use of groups for peer-to-peer support of parents.

Additionally, all the above are challenging points for the NICU to improve the given neonatal care. Based on these implications, the EMPATHIC-N could be completed by parents at the beginning and during their stay at the NICU, in order to assess their needs and provide the best possible care. This way, the EMPATHIC-N could stand as a measure to detect satisfaction change during therapy and would indicate further healthcare needs for policy changes in the NICU. Researchers and clinicians working in neonatal care units in Cyprus should administer the Greek version of the EMPATHIC-N, as it was found to be a reliable instrument assessing different dimensions of parental satisfaction with neonatal care provided.

## Conclusions

To our knowledge, this is the first study that validated the Greek version of the EMPATHIC-N using a Greek-Cypriot population who received services by the only NICU in Cyprus.

Limitations of the present work include the rather small sample that was used for the CFA analyses especially, due to the large number of questionnaire items that did not allow us to have a sample that would be at least ten times higher than the number of questions, as usually suggested. However, the sample was recruited through the NICU which is the most representative unit of the Cypriot population receiving intensive care during one year. Of note, we could not examine potential selection bias among the sample of the present study and the rest of the 384 families who were treated in the NICU but did not participate in this part of the project. It might be that specific characteristics of these families made them different than the rest who gave consent to participate and completed the questionnaire.

Strengths of this work include the methodology used for the translation of the original instrument (forward-backward translation) and the use of confirmatory factor analyses both on the separate subscales and on the whole questionnaire. Future studies should confirm the psychometric properties of the Greek version of the EMPATHIC-N in other Greek-speaking populations (for example in Greece, and in private neonatal care units in Cyprus). The support of the second-order model for the structure of EMPATHIC-N has particular implications concerning the importance of the organizational structure for the parental satisfaction with the care provided by NICUs. The findings of the present study may help the directing team of the NICU in changing strategies and targets.

## Additional file

Additional file 1: EMPATHIC-N original questionnaire items and translated version of items in Greek. (DOCX 37 kb)

## Abbreviations

CFA: Confirmatory Factor Analysis
EMPATHIC-N: Empowerment of Parents in the Intensive Care-Neonatology
NICU: Neonatal Intensive Care Unit
PICU: Pediatric Intensive Care Unit

## References

[1] Als H (2009). NIDCAP: testing the effectiveness of a relationship-based comprehensive intervention. Pediatrics.

[2] American Academy of Pediatrics on Hospital Care and Institute for Patient- and Family-Centered Care. Patient and family-centered care and the pediatrician’s role. Pediatrics 2012;129:394–404. https://doi.org/10.1542/peds.2011-3084 10.1542/peds.2011-3084 22291118

[3] Haumont D. NIDCAP and developmental care. J Pediatr Neonatal Ind Med 2014;3(2):e030240. https://doi.org/10.7363/030240.

[4] Gooding JS, Cooper LG, Blaine AL, Franck LS, Howse JL, Berns SD. Family support and family-centered care in the neonatal intensive care unit: origins, advances, impact. Semin Perinatol 2011;35(1):20–28. https://doi.org/10.1053/j.semperi.2010.10.004 10.1053/j.semperi.2010.10.004 21255703

[5] Cooper LG, Gooding JS, Gallagher J, Sternesky L, Ledsky R, Berns SD. Impact of a family-centered care initiative on NICU care, staff and families. J Perinatol 2007;27: S32-S37. https://doi.org/10.1038/sj.jp.7211840 10.1038/sj.jp.7211840 18034178

[6] Manning AN. The NICU experience how does it affect the parents’ relationship? J Perinat Neonatal Nurs 2012;26:353–357. https://doi.org/10.1097/JPN.0b013e3182710002 10.1097/JPN.0b013e3182710002 23111724

[7] Weissenstein A, Straeter A, Villalon G, Luchter E, Bittman S. Parent satisfaction with a pediatric practice in Germany: a questionnaire-based study. Ital J Pediatr 2011;37:31–37. https://doi.org/10.1186/1824-7288-37-31 10.1186/1824-7288-37-31 PMC3163525 21729322

[8] Carlier IV, Meuldijk D, Van Vliet IM, Van Fenema E, Van der Wee NJ, Zitman FG (2012). Routine outcome monitoring and feedback on physical or mental health status: evidence and theory. J Eval Clin Pract.

[9] Cleveland LM. Parenting in the neonatal intensive care unit. J Obstet Gynecol Neonatal Nurs 2008;37(6):666–691. https://doi.org/10.1111/J.1552-6909.2008.0028 10.1111/j.1552-6909.2008.00288.x 19012717

[10] Conner JM, Nelson EC. Neonatal Intensive Care: Satisfaction measured from a parents’ perspective. Pediatrics 1999;103(Supplement E1):336–349. https://doi.org/10.1542/peds.103.1.SE1.336 9917476

[11] Latour JM, Duovenoorden HJ, Hazelzet JA, van Goudoever JB. Development and validation of neonatal intensive care parent satisfaction instrument. Pediatr Crit Care Med 2012;13(5):554–559. https://doi.org/10.1097/PCC.Ob013e318238b80a 10.1097/PCC.0b013e318238b80a 22460771

[12] Latour JM, van Goudoever JB, Hazelzet JA. Parent satisfaction in the pediatric ICU. Pediatr Clin N Am 2008;55(3):779–790. https://doi.org/10.1016/j.pcl.2008.02.013 10.1016/j.pcl.2008.02.013 18501765

[13] Latour JM, van Goudoever JB, Duivenvoorden HJ, Albers MJ, van Dam NA, Dullaart E, van Heerde M, de Neef M, Verlaat CW, van Vught EM, Hazelzet, JA. Construction and psychometric testing of the EMPATHIC questionnaire measuring parent satisfaction in the pediatric intensive care unit. Intensive Care Med 2011;37(2):310–318. https://doi.org/10.1007/s00134-010-2042-y 10.1007/s00134-010-2042-y PMC3028088 20848078

[14] Ballweg DD (2001). Implementing developmentally supportive family-centered care in the newborn intensive Care unit as a quality improvement initiative. J Perinat Neonat Nurs.

[15] Cheldelin LV, Dunham S, Stewart V. NICU patient satisfaction: how you measure counts. J Perinatol 2013;33(4):324–326. https://doi.org/10.1038/jp.2012.115 10.1038/jp.2012.115 22975982

[16] Dall’Oglio I, Portanova A, Fiori M, Gawronski O, Fida R, Cocchieri A, Rocco G, Tiozzo E, Latour J. Implementing the family-centered care model, parents’ satisfaction and experiences in neonatology. Ital J Pediatr 2014;40(Suppl2):A47. https://doi.org/10.1186/1824-7288-40-S2-A47.

[17] Gomez DB, Vidal SA, Lima LC. Brazilian adaptation and validation of the empowerment of parents in the intensive Care-neonatology (EMPATHIC-N) questionnaire. Jornal de Pediatria. 2016;jjped2016.06.007 (Epub ahead of print). 10.1016/j.jped.2016.06.007 27565641

[18] Butt ML, McGrath JM, Samra HA, Gupta R. An integrative review of parent satisfaction with care provided in the neonatal intensive care unit. J Obstet Gynecol Neonatal Nurs 2013;42(1):105–120. https://doi.org/10.1111/1552-6909.12002 10.1111/1552-6909.12002 23316895

[19] Otani K, Herrmann PA, Kurz RS. Improving patient satisfaction in hospital care settings. Health Serv Manag Res 2011;24(4):163–169. https://doi.org/10.1258/hsmr.2011.011008 10.1258/hsmr.2011.011008 22040943

[20] Azoulay E, Chaize M, Kentish-Barnes N. Involvement of ICU families in decisions: fine-tuning the partnership. Ann Intensive Care 2014;4(1):37–47. https://doi.org/10.1186/s13613-014-0037-5 10.1186/s13613-014-0037-5 PMC4273688 25593753

[21] Guyatt GH, Mitchell A, Molloy DW, Capretta R, Horsman J, Griffith L. Measuring patient and relative satisfaction with level or aggressiveness of care and involvement in care decisions in the context of life threatening illness. J Clin Epidemiol 1995;48(10):1215–1224. https://doi.org/10.1016/0895-4356(95)00024-X 10.1016/0895-4356(95)00024-x 7561983

[22] Aletras VH, Kostarelis A, Tsitouridou M, Niakas D, Nicolaou A. Development and preliminary validation of a questionnaire to measure satisfaction with home care in Greece: an exploratory factor analysis of polychoric correlations. BMC Health Serv Res 2010;10:189–194. https://doi.org/10.1186/1472-6963-10-189 10.1186/1472-6963-10-189 PMC2912895 20602759

[23] Health Monitoring Unit. Perinatal Health Report for the years 2014–2016. https://www.moh.gov.cy/Moh/MOH.nsf/All/8DC461429CBC4DE7C22579CE002EF07D/$file/Perinatal%20Health%20Report%202018_Cyprus%20Maternity%20Units%202014-2016.pdf. Retrieved on 12 Aug 2018.

[24] Dall’Oglio I, Fiori M, Tiozzo E, Mascolo R, Portanova A, Gawronski O, Ragni A, Amadio P, Cocchieri A, Fida R, Alvaro R, Rocco G, Latour JM. Neonatal intensive care parent satisfaction: a multicenter study translating and validating the Italian EMPATHIC-N questionnaire. Ital J Pediatr 2018;44(1):5–14. https://doi.org/10.1186/s13052-017-0439-8 10.1186/s13052-017-0439-8 PMC5756347 29304879

[25] Kline RB (2009). Principles and practice of structural equation modeling.

[26] Hu L, Bentler PM. Cutoff criteria for fit indexes in covariance structure analysis: conventional criteria versus new alternatives. Struct Equ Model Multidiscip J 1999;6(1):1–55. https://doi.org/10.1080/10705519909540118

[27] Hall SL, Ryan DJ, Beatty J, Grubbs L. Recommendations for peer-to-peer support for NICU parents. J Perinatol 2015;35(Suppl.1):S9–S13. http://doi.org/10.1038/jp.2015.143. 10.1038/jp.2015.143 PMC4694192 26597805

