# Supplementary material for: EMPATHIC-N in a Greek-Cypriot sample: confirming its factorial structure
Source: BMC Health Serv Res. 2018 Dec 14;18:968. doi: 10.1186/s12913-018-3793-3 (PMC6295023; doi:10.1186/s12913-018-3793-3)
Supplement: Supplementary file 1 — EMPATHIC-N original questionnaire items and translated version of items in Greek. (DOCX 37 kb) [file 12913_2018_3793_MOESM1_ESM.docx]

Additional file 1: EMPATHIC-N original questionnaire items and translated version of items in Greek.

| Information | **certainly NO** | | | | | |  | |  | |  | |  | | **certainly YES** | | | | **not applicable** | |  |  |  |
| --- | --- | --- | --- | --- | --- | --- | --- | --- | --- | --- | --- | --- | --- | --- | --- | --- | --- | --- | --- | --- | --- | --- | --- |
| We had daily talks about our child’s care and treatment with the doctors and the nurses.  Τυγχάναμε ενημέρωσης σε καθημερινή βάση για την πρόοδο της υγείας του παιδιού μας από το ιατρονοσηλευτικό προσωπικό. | | | | |  | |  | |  | |  | |  | |  | |  | |  | |  | |  |
| Our questions were clearly answered by the doctors and the nurses  Δίνονταν ξεκάθαρες απαντήσεις στα ερωτήματά μας. | | |  | |  | |  | |  | |  | |  | |  | |  | |  | |  |  |  |
| The information given by the doctors and nurses was always the same  Η πληροφόρηση που δινόταν από τους γιατρούς και τους νοσηλευτές ήταν πάντοτε ίδια. | | |  | |  | |  | |  | |  | |  | |  | |  | |  | |  |  |  |
| We were always informed right away when our child’s physical condition worsened  Η ενημέρωση δινόταν σε εμάς αμέσως μετά την επιδείνωση της κλινικής κατάστασης του παιδιού μας. | | |  | |  | |  | |  | |  | |  | |  | |  | |  | |  |  |  |
| We were given clear information about our child’s disease by the doctors and nurses  Λαμβάναμε σαφείς πληροφορίες για το νόσημα του παιδιού μας. | | |  | |  | |  | |  | |  | |  | |  | |  | |  | |  |  |  |
| The doctor clearly informed us about the consequences of our child’s treatment  Ο γιατρός μας πληροφορούσε με σαφήνεια για τις συνέπειες της θεραπείας στο παιδί μας. | | |  | |  | |  | |  | |  | |  | |  | |  | |  | |  |  |  |
| The doctors and nurses gave understandable information about the examinations and tests  Λάβαμε σαφή πληροφόρηση για τις εξετάσεις και τα πραγματοποιούμενα τεστ. | | |  | |  | |  | |  | |  | |  | |  | |  | |  | |  |  |  |
| We received understandable information about the effects of the drugs by the doctors and nurses  Μας δόθηκαν κατανοητές πληροφορίες για τις συνέπειες της φαρμακευτικής αγωγής | | |  | |  | |  | |  | |  | |  | |  | |  | |  | |  |  |  |
| The doctor informed us about the expected health outcomes of our child  Ο γιατρός μας ενημέρωσε για τις μακροπρόθεσμες συνέπειες στη νοσηλεία του παιδιού μας. | | |  | |  | |  | |  | |  | |  | |  | |  | |  | |  |  |  |
| The information brochure we received was complete and clear  Το έντυπο πληροφόρησης που μας δόθηκε ήταν πλήρες και σαφές. | | |  | |  | |  | |  | |  | |  | |  | |  | |  | |  |  |  |
| The information provided by the doctors and nurses was understandable  Οι πληροφορίες που μας δίνονταν από το ιατρονοσηλευτικό προσωπικό ήταν κατανοητές. | | |  | |  | |  | |  | |  | |  | |  | |  | |  | |  |  |  |
| The doctors and nurses gave honest information to us  Το ιατρονοσηλευτικό προσωπικό μας έδινε ειλικρινείς πληροφορίες. | | |  | |  | |  | |  | |  | |  | |  | |  | |  | |  |  |  |
| Care & Treatment | **certainly NO** | | | | | |  | |  | |  | |  | | **certainly YES** | | | | **not applicable** | |  |  |  |
| The doctors and nurses worked closely together  Το ιατρονοσηλευτικό προσωπικό έχει μια στενή συνεργασία. | | |  | |  | |  | |  | |  | |  | |  | |  | |  | |  |  |  |
| The team was alert to the prevention and treatment of pain in our child  Το προσωπικό ήταν ευαισθητοποιημένο για την πρόληψη και θεραπεία του πόνου στο παιδί μας. | | |  | |  | |  | |  | |  | |  | |  | |  | |  | |  |  |  |
| The doctors and nurses are real professionals; they know what they are doing.  Οι γιατροί και οι νοσηλευτές είναι επαγγελματίες, ξέρουν πραγματικά τι κάνουν. | | |  | |  | |  | |  | |  | |  | |  | |  | |  | |  |  |  |
| The correct medication was always given on time  Η ενδεδειγμένη θεραπευτική αγωγή δινόταν πάντα στο σωστό χρόνο. | | |  | |  | |  | |  | |  | |  | |  | |  | |  | |  |  |  |
| At admission, our child’s medical history was known by the doctors and nurses  Κατά την εισαγωγή, το ιατρικό ιστορικό του παιδιού μας ήταν γνωστό στο ιατρονοσηλευτικό προσωπικό. | | |  | |  | |  | |  | |  | |  | |  | |  | |  | |  |  |  |
| Attention was paid to our child’s developmental by the doctors and nurses  Δινόταν έμφαση από το ιατρονοσηλευτικό προσωπικό στην ανάπτυξη του παιδιού μας. | | |  | |  | |  | |  | |  | |  | |  | |  | |  | |  |  |  |
| When our child’s condition worsened, action was immediately taken by the doctors and nurses  Όταν η κατάσταση του παιδιού μας επιδεινώθηκε, έγινε άμεση παρέμβαση από το ιατρονοσηλευτικό προσωπικό. | | |  | |  | |  | |  | |  | |  | |  | |  | |  | |  |  |  |
| Our child’s needs were well taken care of  Λαμβάνονταν υπόψιν οι ανάγκες του παιδιού μας. | | |  | |  | |  | |  | |  | |  | |  | |  | |  | |  |  |  |
| The team had a common goal: the best care and treatment for our child and ourselves  Ο στόχος του ιατρονοσηλευτικού προσωπικού ήταν ένας: η καλύτερη φροντίδα και θεραπεία για το δικό μας παιδί και εμάς. | | |  | |  | |  | |  | |  | |  | |  | |  | |  | |  |  |  |
| Our child’s comfort was taken into account by the doctors and nurses  Η άνεση του παιδιού μας λαμβανόταν υπόψιν από το ιατρονοσηλευτικό προσωπικό. | | |  | |  | |  | |  | |  | |  | |  | |  | |  | |  |  |  |
| Care & Treatment (continued) | | | | | **certainly NO** | | | | | |  | |  | |  | |  | | **certainly YES** | | | | **not applicable** |
| Every day we knew who of the doctors and nurses was responsible for our child  Καθημερινά γνωρίζαμε ποιος γιατρός και νοσηλευτής ήταν υπεύθυνος για το παιδί μας. | | | | | | |  | |  | |  | |  | |  | |  | |  | |  | |  |
| We were emotionally supported by the doctors and nurses  Ενισχυθήκαμε συναισθηματικά. | | | | | | |  | |  | |  | |  | |  | |  | |  | |  | |  |
| The doctors and nurses responded well on our own needs  Οι γιατροί και νοσηλευτές ανταποκρίθηκαν ικανοποιητικά στις δικές μας ανάγκες. | | | | | | |  | |  | |  | |  | |  | |  | |  | |  | |  |
| The team was caring to our child and to us  Η ομάδα νοιαζόταν-φρόντιζε για εμάς και το παιδί μας. | | | | | | |  | |  | |  | |  | |  | |  | |  | |  | |  |
| During acute situations there was always a nurse to support us  Στη διάρκεια οξέων καταστάσεων υπήρχε πάντοτε νοσηλευτής για να μας ενισχύσει. | | | | | | |  | |  | |  | |  | |  | |  | |  | |  | |  |
| Our child was always well taken care of by the nurses while in the incubator/bed  Το παιδί μας πάντοτε ελάμβανε σωστή αγωγή από το νοσηλευτικό προσωπικό στη θερμοκοιτίδα ή στην κούνια. | | | | | | |  | |  | |  | |  | |  | |  | |  | |  | |  |
| The transfer of care from the NICU staff to colleagues in the pediatric ward had gone well  Η μετάβαση της φροντίδας από των θάλαμο εντατικής νεογνών σε ενδιάμεση νοσηλεία ή παιδιατρικό θάλαμο έβαινε ομαλά. | | | | | | |  | |  | |  | |  | |  | |  | |  | |  | |  |
| Parental Participation | | | | | **certainly NO** | | | | | |  | |  | |  | |  | | **certainly YES** | | | | **not applicable** |
| We were actively involved in decision-making on care and treatment of our child  Ήμασταν ενεργά αναμεμειγμένοι στη λήψη αποφάσεων που αφορούσαν τη φροντίδα και θεραπεία του παιδιού μας. | | | | | | |  | |  | |  | |  | |  | |  | |  | |  | |  |
| We were encouraged to stay close to our child  Μας ενθάρρυναν για να παραμείνουμε κοντά στο παιδί μας. | | | | | | |  | |  | |  | |  | |  | |  | |  | |  | |  |
| We had confidence in the team  Είχαμε εμπιστοσύνη στην ομάδα | | | | | | |  | |  | |  | |  | |  | |  | |  | |  | |  |
| Even during intensive procedures we could always stay close to our child  Ακόμα και κατά τις επείγουσες διαδικασίες μπορούσαμε να μείνουμε κοντά στο παιδί μας. | | | | | | |  | |  | |  | |  | |  | |  | |  | |  | |  |
| The nurses stimulated us to help in the care of our child  Το νοσηλευτικό προσωπικό μας ενθάρρυνε στο να συμμετέχουμε στη φροντίδα του παιδιού μας. | | | | | | |  | |  | |  | |  | |  | |  | |  | |  | |  |
| The nurses helped us in the bonding with our child  Το νοσηλευτικό προσωπικό βοήθησε στο να συνδεθούμε συναισθηματικά με το παιδί μας. | | | | | | |  | |  | |  | |  | |  | |  | |  | |  | |  |
| The nurses had trained us the specific aspects of newborn care  Το νοσηλευτικό προσωπικό μας εκπαίδευσε στα ειδικά ζητήματα της νεογνικής φροντίδας. | | | | | | |  | |  | |  | |  | |  | |  | |  | |  | |  |
| Before discharge, the care for our child was once more discussed with us  Προ της εξόδου, μας εξηγήθηκε ακόμα μια φορά η φροντίδα του παιδιού μας. | | | | | | |  | |  | |  | |  | |  | |  | |  | |  | |  |
| Organization | | | | | **certainly NO** | | | | | |  | |  | |  | |  | | **certainly YES** | | | | **not applicable** |
| The NICU made us feel safe  Η Μονάδα Νεογνών μας έκανε να νιώσουμε ασφαλείς. | | | | | | |  | |  | |  | |  | |  | |  | |  | |  | |  |
| Our child’s incubator or bed was clean  Η θερμοκοιτίδα ή η κούνια του παιδιού μας ήταν καθαρή. | | | | | | |  | |  | |  | |  | |  | |  | |  | |  | |  |
| The team worked efficiently  Η ομάδα δούλευε αποτελεσματικά. | | | | | | |  | |  | |  | |  | |  | |  | |  | |  | |  |
| The unit could easily be reached by telephone  Μπορείς εύκολα να επικοινωνήσεις με τη μονάδα τηλεφωνικά. | | | | | | |  | |  | |  | |  | |  | |  | |  | |  | |  |
| There was enough space around our child’s incubator/bed  Υπήρχε αρκετός χώρος γύρω από τη θερμοκοιτίδα του παιδιού μας. | | | | | | |  | |  | |  | |  | |  | |  | |  | |  | |  |
| The NICU was clean  Η Μονάδα Νεογνών ήταν καθαρή. | | | | | | |  | |  | |  | |  | |  | |  | |  | |  | |  |
| Noise in the unit was muffled as good as possible  Ο θόρυβος στη μονάδα περιοριζόταν όσον το δυνατόν περισσότερο. | | | | | | |  | |  | |  | |  | |  | |  | |  | |  | |  |
| There was a nice and friendly atmosphere in the NICU  Υπήρχε μια ζεστή ατμόσφαιρα στη μονάδα νεογνών. | | | | | | |  | |  | |  | |  | |  | |  | |  | |  | |  |
| Professional Attitude | | | | | **certainly NO** | | | | | |  | |  | |  | |  | | **certainly YES** | | | | **not applicable** |
| Nurses and doctors always introduced themselves by name and function  Οι νοσηλευτές και οι γιατροί πάντοτε συστήνονταν με βάση το όνομα και την ιδιότητά τους. | | | | | | |  | |  | |  | |  | |  | |  | |  | |  | |  |
| We received sympathy from the doctors and nurses  Εισπράτταμε τη συμπάθεια του ιατρονοσηλευτικού προσωπικού. | | | | | | |  | |  | |  | |  | |  | |  | |  | |  | |  |
| The team worked hygienically  Η ομάδα δούλευε βάσει κανόνων υγιεινής. | | | | | | |  | |  | |  | |  | |  | |  | |  | |  | |  |
| The team respected the privacy of our child and of us  Η ομάδα σεβόταν την ιδιωτικότητα του παιδιού μας και εμάς. | | | | | | |  | |  | |  | |  | |  | |  | |  | |  | |  |
| The team showed respect for our child and for us  Η ομάδα ένιωθε σεβασμό για μας και το παιδί μας. | | | | | | |  | |  | |  | |  | |  | |  | |  | |  | |  |
| At our bedside, the discussion between the doctors and nurses was only about our child  Πλάι στη θερμοκοιτίδα η συζήτηση μεταξύ των γιατρών και των νοσηλευτών αφορούσε μόνον το παιδί. | | | | |  | |  | |  | |  | |  | |  | |  | |  | |  |  |  |
| There was a pleasant atmosphere among the staff  Υπήρχε μια ευχάριστη ατμόσφαιρα διαμέσου του προσωπικού. | | | | | | |  | |  | |  | |  | |  | |  | |  | |  | |  |
| We felt welcomed by the team  Νιώσαμε ότι είμαστε ευπρόσδεκτοι από την ομάδα. | | | | | | |  | |  | |  | |  | |  | |  | |  | |  | |  |
| In spite of the workload, sufficient attention was paid to our child and to us by the team  Παρά το φόρτο εργασίας, δινόταν ικανοποιητική προσοχή σε μας και το παιδί μας από την ομάδα. | | | | |  | |  | |  | |  | |  | |  | |  | |  | |  |  |  |
| Our cultural background was taken into account  Ελήφθη υπόψιν η κουλτούρα μας. | | | | | | |  | |  | |  | |  | |  | |  | |  | |  | |  |
| Our child’s health always came first for the doctors and nurses  Η υγεία του παιδιού μας ήταν πρωταρχικής σημασίας για το ιατρικό και νοσηλευτικό προσωπικό. | | | | | | |  | |  | |  | |  | |  | |  | |  | |  | |  |
| The doctors and nurses always took time to listen to us  Οι γιατροί και οι νοσηλευτές πάντα μας έδιναν χρόνο για να μας ακούσουν. | | | | | | |  | |  | |  | |  | |  | |  | |  | |  | |  |
